# The Relationship Between the Dark Triad Personality Traits, Motivation at Work, and Burnout Among HR Recruitment Workers

**DOI:** 10.3389/fpsyg.2019.01290

**Published:** 2019-06-07

**Authors:** Monika Prusik, Michał Szulawski

**Affiliations:** ^1^ Department of Social Psychology, Faculty of Psychology, University of Warsaw, Warsaw, Poland; ^2^ The Maria Grzegorzewska University, Warsaw, Poland

**Keywords:** dark triad, narcissism, psychopathy, Machiavellianism, intrinsic motivation, extrinsic motivation, burnout

## Abstract

We focus on the Dark Triad personality traits (Machiavellianism, narcissism, and psychopathy) and their relationships to the mechanisms of motivation and level of burnout that people experience at work. From the motivational perspective, the needs associated with the Dark Triad traits might be satisfied in work environments by selecting different goals or motives. Moreover, the selection of different goals and motives may be related to the level of burnout syndrome that some people develop. We use the Short Dark Triad Personality Test, Barbuto’s Motivation Sources Inventory, and Oldenburg Burnout Inventory to measure triad traits, preferred work motives, and level of burnout, respectively. The results show that in general, some part of the relationship between the Dark Triad traits and burnout is mediated by the motivational sources. As expected, the Dark Triad traits are more closely related to external sources of motivation (especially instrumental motivation), which are in turn partly associated with higher levels of burnout. The results also suggest that the trajectory of the relationship between the Dark Triad traits and burnout *via* motivation sources is different from expected, presenting a background for discussion.

## Practitioner Points

- People high in the Dark Triad traits in “HR environment” are motivated at work both by external and internal types of rewards.
- Employees with high levels of narcissism in “HR environment” are highly motivated and resilient to burnout.

## Issue

Recent years have seen a growing body of research on dark traits of personality (Machiavellianism, narcissism and psychopathy) in workplace environments. Their role has primarily been explored in the context of organizational behavior, including effectiveness (Furnham et al., 2012), counterproductive work behavior (O’Boyle et al., 2012; Cohen, 2016), unethical behaviors (Amernic and Craig, 2010), organizational citizenship behavior (Becker and Dan O’Hair, 2007), job crafting (Roczniewska and Bakker, 2016), and skillful leadership and management (Paunonen et al., 2006; Ames, 2009), albeit not in the context of occupational health. For example, beyond a few studies testing direct relationships between job burnout and single dark traits, e.g., narcissism (Schwarzkopf et al., 2016) or psychopathy (Johnson et al., 2015), the relationship between dark traits and job burnout has not been broadly investigated. The present research addresses this literature gap. Aside from testing the direct link between dark traits and job burnout, the mediational effect is examined here. Based on some previous studies, it can be expected that employees who differ in dark traits are guided by different motives at work (Jonason et al., 2014, 2015). Furthermore, a few recent studies have examined the relationship between motivational regulations at work and job burnout (Rawolle et al., 2016; Fernet et al., 2017). However, the obtained results are inconsistent. The current study thus seeks to examine the direct effect of dark traits of personality on job burnout and the mediation effect of work motivation in the Dark Triad-job burnout link.

## Dark Traits and Job Burnout

The majority of research on “dark personality” has focused on three traits that are commonly described as the Dark Triad: Machiavellianism, subclinical narcissism, and subclinical psychopathy (Paulhus and Williams, 2002). Machiavellianism is characterized by cynical, pragmatic, misanthropic, and immoral beliefs; emotional detachment; agentic and self-serving motives; strategic long-term planning; manipulation and exploitation (Christie and Lehman, 1970; Rauthmann and Will, 2011). Narcissism includes an inflated view of self, fantasies about control, success, and admiration, and the desire to have self-love reinforced by others (Kernberg, 1989; Morf and Rhodewalt, 2001). Psychopathy is marked by a lack of concern for both other people and social regulatory mechanisms, impulsivity, and a lack of guilt or remorse for harming others (Hare and Neumann, 2009). Although many researchers have treated the constructs comprising the Dark Triad as singular traits (e.g., psychopathy), it is more appropriate to conceptualize these constructs as multidimensional, being composed of multiple attributes (Cohen, 2016). Indeed, despite overlap between the traits constituting the Dark Triad, they are nonetheless relatively independent (Paulhus and Williams, 2002). The research scope of the present study encompasses three different traits.

Job burnout is defined here as a long-term effect of chronic work-related stress caused by excessive job demands and insufficient job resources (Schaufeli and Bakker, 2004). Initially, the phenomenon of job burnout was conceptualized as pertaining exclusively to human services (e.g., doctors, nurses, teachers). However, more recent research has demonstrated that other occupations – including managers, sales representatives, IT specialists, and soldiers – also manifest symptoms of job burnout (Lee and Ashforth, 1996; Demerouti et al., 2001). In this study, we apply a two-dimensional definition of job burnout (Demerouti et al., 2001), suggesting that it consists of exhaustion and disengagement from work. Exhaustion is a response to intensive physical, affective, and cognitive strain, and is exhibited in fatigue, weariness, and reduced energy. Disengagement is expressed by distancing oneself from work and by presenting negative attitudes toward the whole work-related context, such as duties, workers’ values, and organizational culture. Thus, disengagement is a broader notion that comprises both depersonalization and lack of personal achievement (Demerouti et al., 2001).

The direct link between some dark traits and job burnout has been examined in a few previous studies (Johnson et al., 2015; Schwarzkopf et al., 2016). One found that a high level of narcissism predicts the two components of job burnout: emotional exhaustion and cynicism (Schwarzkopf et al., 2016). In other studies, narcissism has been positively correlated with school burnout among college students (Barnett and Flores, 2016), but generally negatively correlated with stress responses (Richardson and Boag, 2016), and positively correlated with task-oriented coping (Birkás et al., 2016). The relationship between psychopathy and job burnout (or job stress) is less clear. Some authors have not found a significant connection between psychopathy and job stress (Beutler et al., 1988; Bartol et al., 1992; Richardson and Boag, 2016). However, one piece of novel research supports the notion that psychopathy is associated with high rates of job stress and emotional exhaustion, and negative affective experiences (Noser et al., 2014; Johnson et al., 2015). Given that Machiavellianism is related to high job stress (Heisler and Gemmill, 1977; Richardson and Boag, 2016) and low job satisfaction (Jonason et al., 2015), and negatively associated with task-oriented coping strategies, a positive link between Machiavellianism and job burnout may be expected. Overall, relying on the above studies, a positive relationship between the Dark Triad and job burnout is expected.

## Dark Triad and Motivation At Work

The relationship between the Dark Triad and burnout might also be studied from the perspective of motivation. Different work goals or motives in the work environment can be selected or pursued because they satisfy needs or values associated with specific Dark Triad traits (Rosenthal and Pittinsky, 2006; Harrison et al., 2016; Jonason and Ferrell, 2016; Pilch and Górnik-Durose, 2016). For example, a high level of the Dark Triad might result in seeking roles and situations in which one can exploit others (Machiavellianism), pursue recognition and appreciation (narcissism), or find risky situations and stimulations (psychopathy). Among the different models of motivation, the Integrative Taxonomy of Motivation has proved valid in determining a variety of different sources of motivation of employees (Leonard et al., 1995; Barbuto and Scholl, 1998). Barbuto’s model integrates intrinsic/extrinsic, internal/external self-concept and goal internalization traditions to identify the following five basic forms of motivation at work: (1) intrinsic process, (2) internal self-concept, (3) instrumental motivation, (4) external self-concept, and (5) goal internalization. Intrinsic process takes place when a person is engaged in certain kinds of work for the sheer fun of it (Deci and Ryan, 2002). Internal self-concept motivation concerns situations in which an individual engages in behaviors that reinforce the internal standards of traits, competencies, or values that form the basis of the ideal self. Instrumental motivation is fuelled by extrinsic outcomes, such as pay, promotion, and rewards. External self-concept concerns situations in which an individual is primarily other-directed, that is, engages in work behaviors that satisfy reference group members in order to gain acceptance and status. Finally, goal internalization is the adoption of attitudes and behaviors that are congruent with an individual personal value system (Barbuto and Scholl, 1998; Barbuto, 2001).

Predictions that the Dark Triad traits are related to motivational sources can be seen in some recent studies. For example, all three Dark Triad traits are powerful psychological antecedents of fraud behavior (Harrison et al., 2016) and motives of achievement and power (Jonason and Ferrell, 2016). Furthermore, narcissism and Machiavellianism are strong positive predictors of materialism (Rosenthal and Pittinsky, 2006; Pilch and Górnik-Durose, 2016). Machiavellianism and psychopathy, on the other hand, are associated with looking for competition at work (Jonason et al., 2015). Machiavellianism, moreover, is associated with lower engagement in organizational activities and lower teamwork (Kessler et al., 2010; Jonason and Webster, 2012). On the other hand, narcissism has proven to be linked with affiliation and self-affirmation needs (Pilch and Górnik-Durose, 2016). Furthermore, it has been shown that psychopathy is related to risking other people’s money (Jones, 2014), a preference toward realistic, hands-on jobs that do not involve helping others (Morf and Rhodewalt, 2001; Jonason et al., 2014), and to rebelling against authority and engaging in illegal activities (O’Boyle et al., 2012).

A recent study has found that narcissistic, Machiavellian, and psychopathic people vary in their needs and respond aggressively in different situations (Palmer et al., 2017). Specifically, individuals high in narcissism respond more aggressively to ego-threat, whereas psychopaths respond more aggressively when provoked. In Machiavellian personalities, aggression is aggravated when such people are unable to fulfill their own objectives.

## Motivation At Work and Burnout

The direct relationship between motivation and burnout has a solid empirical basis (Cresswell and Eklund, 2005; Van den Broeck et al., 2008; Lonsdale et al., 2009; Li et al., 2013; Van den Berghe et al., 2014). For example, in research conducted by Cresswell and Eklund (2005), amotivation – the least self-determined type of motivation in self-determination theory – presented a strong positive association with burnout and externally regulated motivation, as well as a nonsignificant relationship with burnout. Moreover, self-determined forms of motivation exhibited significant negative associations with burnout. The satisfaction of three basic psychological needs of self-determination theory, associated with building intrinsic motivation, fully explained the relationship between job resources and exhaustion (Van den Broeck et al., 2008). In the study by Lonsdale et al. (2009), autonomy and competence together with self-determined motivation accounted for significant amounts of variance in athlete burnout symptoms. The results of a meta-analysis of three basic psychological needs and motivational regulations and burnout showed that the three universal psychological needs, intrinsic motivation, and extrinsic autonomous regulation had small to large negative effects on predicting burnout (Li et al., 2013). In another study, teachers motivated intrinsically or by integrated forms of extrinsic motivation experienced fewer burnout symptoms (Van den Berghe et al., 2014).

As a consequence of the literature review, we expect that the mechanisms for developing burnout among people with the Dark Triad traits can be explained by the motives they pursue at work and the possibilities of their realization. Given that Dark Triad traits stimulate people to look for external motives at work, we expect that they will constitute the main mechanisms causing burnout. The higher the level of Dark Triad traits, the higher the desire to obtain external kinds of rewards at work, and these, on the other hand, will cause burnout. The other mechanisms of developing burnout will be through internal motives. If internal motives happen to be important for people with high levels of Dark Triad traits (which may be true among HR recruiters), they should decrease the burnout symptoms. More specifically, people high in psychopathy and Machiavellianism should suffer from more burnout symptoms, as we expect that they will not be motivated by internal factors. In contrast, narcissists in Human Resources (HR) recruitment may additionally be internally motivated, and as a consequence less burnt out.

## The Present Study

The present study was conducted with a group of recruiters working either internally at companies’ HR departments or in external retained or contingency recruitment agencies. Recruitment specialists seek candidates for specific jobs, evaluate their experience and competence, and conduct interviews. The HR recruiters group is especially interesting for the purpose of this study, as the profession requires constant contact with different groups of people, including managers and clients, who have varied expectations. In such work contexts, Machiavellians, narcissists, and psychopaths have numerous opportunities to fulfill their goals and motives. In order to evaluate the Dark Triad traits, we used the Dark Triad of Personality Test (D3-Short) (Jones and Paulhus, 2014); to measure the motives pursued at work, we used Barbuto’s Motivation Sources Inventory (Barbuto and Scholl, 1998); and to measure the level of burnout, we used the Oldenburg Burnout Inventory (OLBI) (Demerouti et al., 2001).

Based on the aforementioned literature, we expect that the Dark Triad traits will predict the development of particular sources of motivation at work, and both of these groups of factors will be to some extent related to burnout syndrome among HR recruiters. We also expect that the relationship between the Dark Triad personality traits and burnout will be mediated by motivational factors.

We specifically predict that:

1. The Dark Triad traits are related to job burnout (H1);
  Machiavellianism and psychopathy are positively related to burnout (H1a and H1b);
  Even though some studies report a positive relationship between narcissism and burnout, due to the specificity of the research group we leave the hypothesis open. People high in narcissism may in a profession involving constant interpersonal contact find numerous coping strategies, and consequently not suffer from burnout symptoms (H1c).
2. The Dark Triad traits are positively related to external sources of motivation (e.g., instrumental motivation) (H2);
3. Narcissism is positively related to external self-concept (H3);
4. The Dark Triad traits are related to the internal process of motivation (H4);
  This may be especially true in jobs that require constant contact with people, as managing and persuading others might be central, hence these aims may be achieved by means of manipulation, adoration and mischief.
5. Internal sources of motivation are negatively related to burnout (H5);
6. External sources of motivation are positively related to burnout (H6);
  These hypotheses have mostly been proven by researchers in the self-determination theory framework. We believe that they will also be true in the current study.
7. The relationship between the Dark Triad traits and burnout will be mediated by different forms of motives at work (H7).

We believe that (H7a) external motives will be the main mediator between the Dark Triad traits and burnout. However, the role of external motives is uncertain. The literature states that external motives are more rewarding for workers high in the Dark Triad traits, but they are also positively related to burnout. Consequently, as external motives are desired by workers with greater Dark Triad traits, they might serve as factors protecting them from burnout, as they “get what they want” from their job. On the other hand, external motives can be rewarding in the short term, but cause burnout in the long term. We also predict (H7b) that internal motives will mediate between the Dark Triad traits and burnout. Due to the nature of the job, higher scores on the Dark Triad traits in the group of recruiters might also be related to intrinsic motivation. For example, we expect that the need of entitlement, which is specific to narcissism, might be fulfilled in a recruitment job by that position offering some amount of power over candidates, and by the workers serving as an information hub between managers and clients. These activities may reinforce the internal standards of narcissism, thereby being related to the motive of internal concept. However, internal concept in the case of narcissism might also serve as a protective factor, first because it is one of the internal types of motivation, and second because it is congruent with what a narcissistic worker might seek from a job. Given that there are different possible predictions regarding the mediating role of motivational sources, we leave the hypothesis open.

### Method

#### Participants

Participants comprised recruiters from internal and external HR departments (*N* = 175, 75% women). Women were overrepresented in the study, *χ*^2^(1) = 32.14, *p* < 0.001. The minimum age was 22 and the maximum was 52 years (*M* = 28.86, SD = 5.31). Most of the participants were well educated (63.4% holding a bachelor’s or master’s degree). The distribution of education levels was not equal with underrepresentation of the lower levels of education, *χ*^2^(5) = 309.42, *p* < 0.001. Most participants in the study were also at the beginning of their professional careers, with average seniority at current workplace of 2.10 years (SD = 2.64) and average general seniority of 5.49 years (SD = 4.97). The sample selection was of a non-probability sampling character, and its aim was to reach a specific professional group (recruiters). Potential participants in the study were invited mostly through LinkedIn. The data were complete, with no missing data occurrence.

### Materials

The main questionnaire consisted of an introductory part, a set of sociodemographic questions, and three psychological questionnaires compiled into one larger tool, administered in the following order:

#### Motivation Source Inventory

This questionnaire seeks to measure five types of motivation sources in the form of: intrinsic process (e.g., “I only like to do things that are fun”), internal self-concept (e.g., “I consider myself a self-motivated person”), instrumental motivation (e.g., “I like to keep looking for better business opportunities”), external self-concept (e.g., “I work harder on a project if public recognition is attached to it”), and goal internalization (e.g., “I would not work for a company if I didn’t agree with its mission”) (Barbuto and Scholl, 1998). The first two motivation sources are related to intrinsic motives, and the remaining three are deemed characteristic of external types of motivation. The questionnaire consists of 30 Likert-type items (from 0 – completely disagree to 6 – completely agree, no items were reversed), with six for every measured subscale. In our study, the Cronbach’s alphas for the five subscales were good or very good and ranged between 0.69 and 0.81. In statistical analysis, we used indicators based on averaged scores.

#### Dark Triad of Personality

This is a brief measure of traits related to the Dark Triad personalities. The questionnaire consists of 27 Likert-type items (the range of the scale used was from 1 – completely disagree to 5 – completely agree, five items were reversed) (Jones and Paulhus, 2014). An equal number of items is dedicated to measure Machiavellianism (e.g., “Make sure your plans benefit you, not others”), narcissism (e.g., “I know that I am special because everyone keeps telling me so”), and psychopathy (e.g., “People who mess with me always regret it”). It is necessary to stress that the diagnosis is subclinical. In our study, Cronbach’s alphas ranged from 0.71 to 0.76 (0.86 for the overall scale). Indicators based on averages were saved and used for further analysis.

#### Oldenburg Burnout Inventory

The Polish version was prepared by Cieślak (Demerouti et al., 2001, 2003; Baka and Cieślak, 2010). The questionnaire consists of 16 Likert-type items (on a scale from 1 – agree to 4 – disagree, some items are reversed). The tool measures two types of burnout: disengagement (eight items, e.g., “Lately, I tend to think less at work and do my job almost mechanically”) and exhaustion (e.g., “After my work, I usually feel worn out and weary”). The instrument has good reliability coefficients (*α* = 0.73 for disengagement and *α* = 0.77 for exhaustion), and its validity has been examined previously (Baka, 2011; Baka and Basińska, 2016, *N* = 2,216). In further analysis, we used indicators in the form of averaged results.

### Procedure

Potential participants in the study were sent a link to an Internet-based questionnaire. The questionnaire had an introductory part in which all participants were informed about the nature, purpose, and confidentiality of the gathered data. Participants in the study expressed their consent by returning the completed questionnaire. Participation in the study was voluntary and no gratification was given. Time for completion of the questionnaire was not limited, but the average time spent on completion was 25 min (SD = 4.20). The data were collected within a research project conducted at The Maria Grzegorzewska University (Malanowska and Prusik, 2016). Ethics committee approval was not required for the study as per applicable institutional and national guidelines and regulations, and the informed consent of the participants was implied through survey completion.

## Results

First, we examined the relationship between the studied constructs by calculating the Pearson correlation (*r*) coefficients (Table 1). All constructs were normally distributed based on graphical inspection of histograms and values of skewness and kurtosis falling into the acceptable range of <−1, 1>. We also checked linearity assumption by inspection of scatterplots for randomly chosen pairs of variables. According to our findings, all three groups of constructs were positively intercorrelated (the Dark Triad measures, motivational sources, burnout types). More specifically, Machiavellianism and psychopathy were positively related to both types of burnout. However, narcissism was not related to either form of burnout. The Dark Triad measures were also related to motivational sources in various configurations. In general, all Dark Triad measures were positively related to intrinsic process and instrumental motivation. However, narcissism was also positively related to internal and external self-concept as well as goal internationalization (in fact, to all measured motivation types). Machiavellianism was also positively associated with external self-concept. As for motivational sources, only the relationship between internal self-concept and disengagement was statistically significant, and it was negative. Intrinsic process and instrumental motivation were positively associated with exhaustion. The correlational analysis results provided a good basis to test the mediational effects of the studied constructs.

**Table 1:** Bivariate correlations, means and standard deviations of the main constructs, *N* = 175.

			Dark Triad			Motivational sources					Burnout	
	*M*	SD	1	2	3	4	5	6	7	8	9	10
Machiavellianism	3.08	0.60	–									
Narcissism	3.05	0.53	**0.34** ^***^	–								
Psychopathy	2.19	0.56	**0.70** ^***^	**0.43** ^***^	–							
Intrinsic process	4.37	0.68	**0.27** ^***^	**0.29** ^***^	**0.18** ^*^	–						
Internal self-concept	4.60	0.64	0.12	**0.38** ^***^	0.08	**0.41** ^***^	–					
Instrumental motivation	4.30	0.85	**0.36** ^***^	**0.35** ^***^	**0.31** ^***^	**0.42** ^***^	**0.39** ^***^	–				
External self-concept	4.60	0.76	**0.23** ^**^	**0.34** ^***^	0.07	**0.44** ^***^	**0.60** ^***^	**0.59** ^***^	–			
Goal internalization	4.56	0.76	0.04	**0.22** ^**^	−0.04	**0.26** ^**^	**0.62** ^***^	**0.34** ^***^	**0.38** ^***^	–		
Disengagement	2.24	0.49	**0.23** ^**^	−0.07	**0.25** ^**^	0.13	**−0.18** ^*^	0.13	−0.02	−0.11	–	
Exhaustion	2.30	0.53	**0.23** ^**^	−0.03	**0.29** ^***^	**0.17** ^*^	−0.14	**0.22** ^**^	0.08	−0.09	**0.74** ^***^	–

* p < 0.05;

** *p < 0.01;*

*** *p < 0.001*.

We also checked the relationship between study constructs, basic sociodemographic characteristics, and measures related to work by calculating Spearman’s correlation coefficients (rho), as some of the sociodemographic variables were not perfectly normally distributed (slightly too skewed), such as age (Table 2).

**Table 2:** Spearman’s rho correlations coefficients of the main constructs, sociodemographic variables and measures related to work, *N* = 175.

	Dark Triad			Motivation sources					Burnout	
	Mach.	Nar.	Psych.	Intrinsic process	Internal self-concept	Instrumental motivation	External self-concept	Goal internalization	Disengagement	Exhaustion
Gender (0 – M, 1 – F)	**−0.19** ^*^	−0.05	**−0.16** ^*^	0.00	0.02	−0.04	0.13	0.07	−0.03	**0.15** ^*^
Age	−0.11	−0.07	−0.07	−0.13	−0.09	−0.11	−0.12	−0.05	−0.11	**−0.16** ^*^
Education	−0.12	−0.01	−0.08	**−0.16** ^*^	−0.05	−0.10	−0.08	0.00	−0.04	**−0**.11
Standard of living	−0.11	0.13	−0.06	−0.06	0.02	−0.10	−0.06	−0.05	**−0.28** ^**^	**−0.27** ^**^
Place of work (0-internal HR, 1-external HR)	**0.20** ^**^	−0.01	0.13	0.13	0.03	**0.17** ^*^	0.02	0.14	**0.16** ^*^	**0.20** ^**^
Seniority – general	−0.12	0.01	−0.09	**−0.18** ^*^	−0.05	−0.10	−0.08	−0.08	**−0.16** ^*^	**−0.21** ^**^
Seniority – at current workplace	0.07	0.01	0.05	−0.14	−0.03	−0.03	−0.02	0.04	0.08	−0.03
Hours of work weekly	0.08	−0.12	0.11	−0.07	−0.13	−0.06	−0.11	−0.12	0.10	0.18^*^

* *p < 0.05;*

** *p < 0.01;*

*** *p < 0.001*.

Overall, the studied constructs were not closely related to one another (if anything, there were small correlation coefficients). Dark Triad measures (besides narcissism) were higher among men than women. Exhaustion levels were higher among women. Standard of living, age (only for exhaustion), and general seniority were negatively related to burnout intensity. Work in external HR rather than internal HR department was related to higher Machiavellianism, instrumental motivation, and higher tendency toward disengagement and exhaustion. Higher general seniority and higher levels of education coincided with lower levels of intrinsic process.

**Figure 1:**
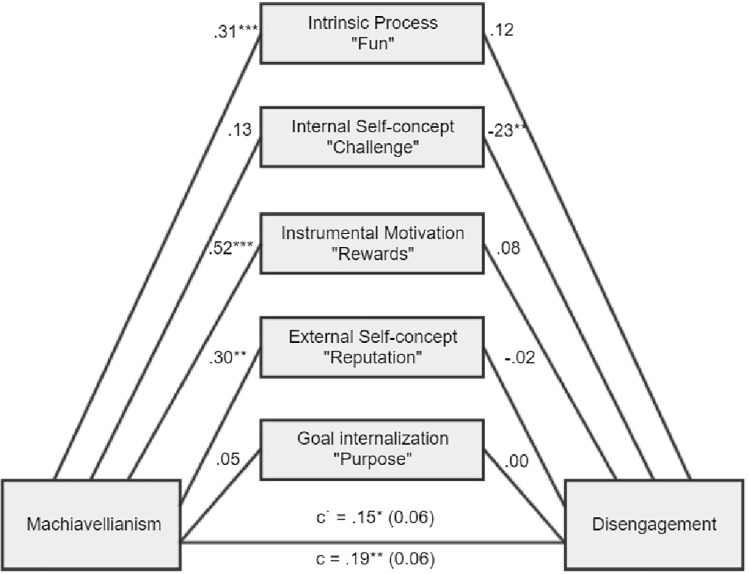
There was a nonsignificant total indirect effect of ab = 0.04, BCa CI (−0.03, 0.12). There was a significant indirect effect for intrinsic process, ab = 0.04, BCa CI (0.01, 0.09).

**Figure 2:**
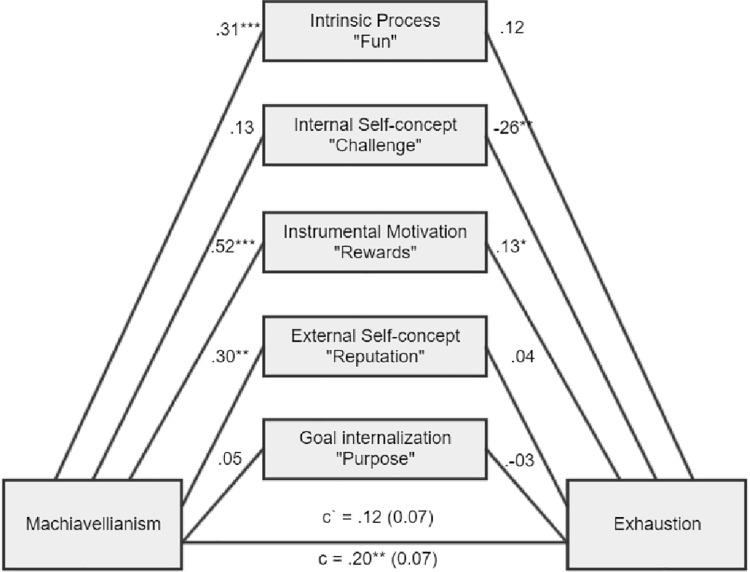
There was a significant total indirect effect of ab = 0.08, BCa CI (0.01, 0.17). There was a significant indirect effect for intrinsic process, ab = 0.04, BCa CI (0.01, 0.10), and for instrumental motivation, ab = 0.07, BCa CI (0.01, 0.15).

**Figure 3:**
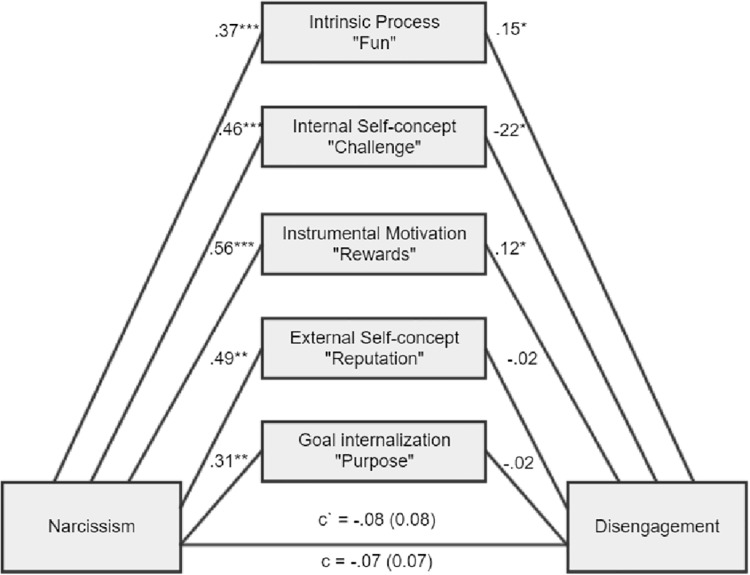
There was a nonsignificant total indirect effect of ab = 0.01, BCa CI (−0.10, 0.10). There was a significant indirect effect for intrinsic process, ab = 0.05, BCa CI (0.02, 0.12), internal self-concept, ab = −0.10, BCa CI (−0.21, −0.02), and instrumental motivation, ab = 0.07, BCa CI (0.02, 0.14).

**Figure 4:**
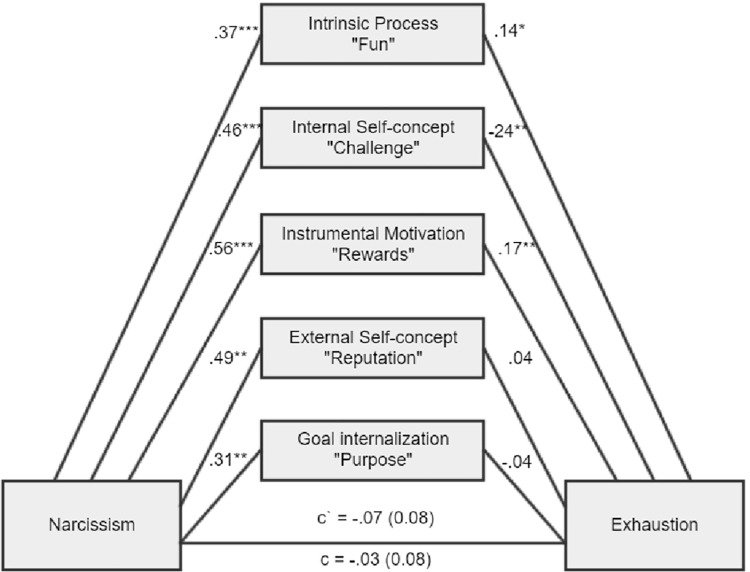
There was a nonsignificant total indirect effect of ab = 0.04, BCa CI (−0.07, 0.15). There was a significant indirect effect for intrinsic process, ab = 0.05, BCa CI (0.01, 0.13), internal self-concept, ab = −0.11, BCa CI (−0.24, −0.03), and instrumental motivation, ab = 0.10, BCa CI (0.02, 0.20).

**Figure 5:**
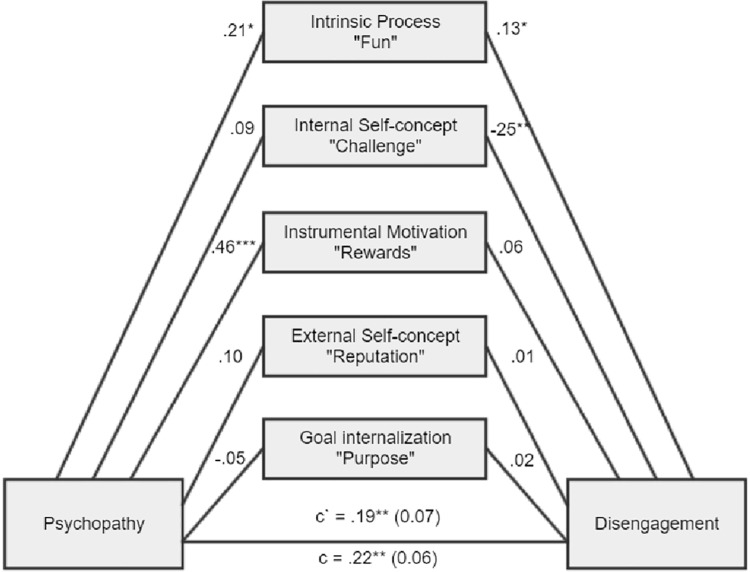
There was a nonsignificant total indirect effect of ab = 0.03, BCa CI (−0.03, 0.10). There was a significant indirect effect for intrinsic process, ab = 0.03, BCa CI (0.00, 0.08).

**Figure 6:**
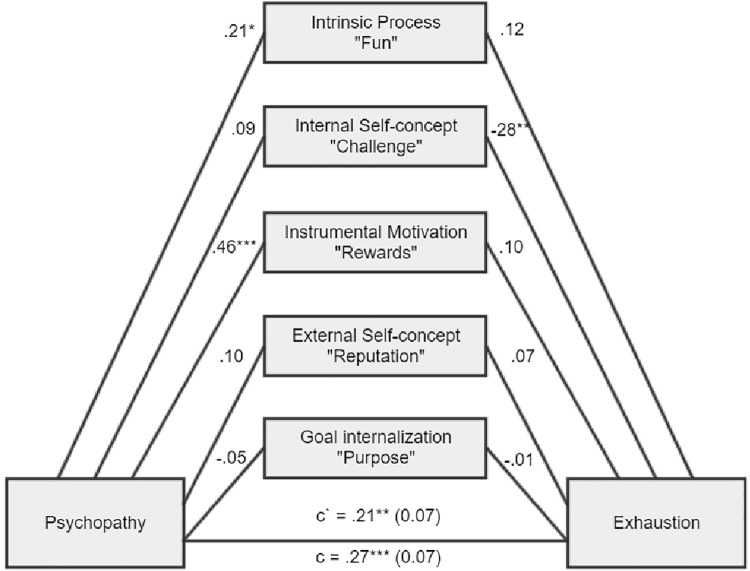
There was a nonsignificant total indirect effect of ab = 0.06, BCa CI (−0.03, 0.13). There was a significant indirect effect for intrinsic process, ab = 0.03, BCa CI (0.00, 0.08).

We decided to proceed with a mediational analysis, whose design and results are presented in Figures 1–6, Supplementary Table S1A.

As expected, Machiavellianism and psychopathy in general exert burnout tendencies in both forms (disengagement and exhaustion). Two main hypotheses were confirmed (path c – significant total effects, H1a and H1b). However, it appears that narcissism is not related to burnout at all (H1c), while this is true of Machiavellianism and psychopathy (H1a, H1b confirmed). Furthermore, the relationships between the Dark Triad traits and various types of motivational sources are mostly confirmed (H2, H3, and H4). Particular Dark Triad traits are all positively related to intrinsic process and instrumental motivation (paths a1 and a3). However, narcissism is also related to the remaining sources of motivation (paths a2, a4, a5), and Machiavellianism is related to external self-concept (path a3 sig.). Judging by the effects between motivation sources and burnout, we can see that two types of internal motives are particularly related to burnout (H5). Intrinsic process is positively related to disengagement and exhaustion (some of the paths b1 sig.), while internal self-concept is negatively related to disengagement and exhaustion (paths b2 sig.). Moreover, instrumental motivation is positively related to disengagement and exhaustion, but only for a model with narcissism (paths b3 sig.; H6). In addition, instrumental motivation is positively related to exhaustion, but for a model with Machiavellianism. Regarding the total indirect effects, only one was significant (Machiavellianism leading to exhaustion *via* sources of motivation, paths a1–a5 × b1–b5; H7). However, many of the individual indirect effects (involving particular types of motivational sources) played a significant role. Altogether, taking into account indirect, direct, and total effects and their significance or lack thereof for the six tested mediations, when concentrating only on significant results, we can state the following: (1) stronger intrinsic process partially mediated the Machiavellianism and disengagement connexion; (2) the relationship between Machiavellianism and exhaustion was fully mediated by sources of motivation, but primarily due to the role of intrinsic process and instrumental motivation; (3) narcissism in general was not related to any type of burnout, so it is not possible to speak here of mediational effects; (4) stronger intrinsic process partially mediated psychopathy and disengagement relation; (5) stronger intrinsic process also partially mediated the psychopathy-exhaustion relationship.

## Discussion

Burnout is a multidimensional construct whose consequences can assume various forms, including the most pronounced impairment of professional functioning. One of the aims of this study was to go beyond the commonly referenced sources, consequences, and symptoms of burnout, and to concentrate on burnout triggers in the form of motivations, especially subclinical pathological forms of personality traits. Dark Triad traits were of particular interest here. Numerous studies have been dedicated to the Dark Triad traits, but few to our knowledge concentrate specifically on the link between Dark Triad personality traits and occupational health (or rather its poor state) in the form of burnout. Furthermore, the paths between the Dark Triad traits and motivations were examined. We believe that our research has filled some of the gaps in the literature. We have formed several hypotheses on the nature of the relationship between the Dark Triad traits, motivational sources, and burnout in the form of exhaustion and disengagement. Our hypotheses were specific (related to one link at a time between two constructs in the forms of paths a, b, c) as well as mediational (gathering together three constructs of the Dark Triad traits, motivation sources, and burnout).

In general, the results showed a significant relationship between burnout and the Dark Triad traits (except for narcissism; thus, H1 was partly confirmed). They mostly corroborated the idea that the Dark Triad traits should be examined separately, as each resulted in different characteristics of the relationship between motivation sources and burnout. Of course, these results can only be interpreted within the specific context of the study (HR recruiters). On the other hand, the results not only have theoretical value, but also an applicable benefit. Based on our results, each group of the Dark Triad traits can now be characterized in relationship to burnout and motivations.

According to our results, narcissists are highly motivated people who do not burn out easily. Narcissism is related to each of the motivations examined. The Dark Triad traits are usually associated with mostly external, instrumental motivations. In this case, a higher level of narcissism was related to both internal and external motivations. This confirms hypotheses H2, H3, and H4, but also goes beyond our expectations, and shows the significant link between narcissism and intrinsic (fun at work) types of motivation. Narcissism was not associated with disengagement and exhaustion (H1 not confirmed for narcissism). This accords with previous research results in which narcissism usually stands out as the “brightest” Dark Triad member, while Machiavellianism and psychopathy have more detrimental effects (e.g., Rauthmann and Kolar, 2013; Volmer et al., 2016). However, the conclusion that narcissism might represent a desirable trait should not be so easily drawn. Narcissists, although motivated and resistant to burnout, also exhibit undesirable features (not examined here) that might pose problems in any workplace where empathy, care, and concern for others are desired: practically everywhere. For example, narcissism coincides with a tendency to use a whole array of manipulation techniques at work (causing narcissists to be considered toxic employees) (Jonason et al., 2012), unethical conduct (Amernic and Craig, 2010), and numerous other previously mentioned undesirable behaviors toward others. Thus, our results present only part of the picture. Nevertheless, narcissism as it appears in our study is related to having fun at work and a sense of company purpose, but also to preoccupation with money, rewards, and reputation, as well as finding one’s work challenging. None of the motivation types seems to dominate the picture; at most, perhaps there is a slight predomination of instrumental motivation, internal and external self-concept over intrinsic process and goal internalization. As predicted, in the model with narcissism, external motivations (but only in the form of instrumental motivation) were related to higher burnout tendencies, which partly confirms hypothesis H6. However, the relationship between internal motivations and narcissism has a more complicated character. In accordance with our assumptions, internal self-concept was related to lower tendency to burnout (supports H5), but intrinsic process was linked to higher tendency to burnout (disconfirms H5).

In contrast to narcissism, which is generally associated with high motivation and low burnout, psychopathy among recruiters is associated with varied forms of motivation and levels of burnout. A result congruent with our main hypothesis is that instrumental motivation is the strongest motivator for recruiters who are high in psychopathy (H2 confirmed). However, this type of motivation does not mediate the relationship between psychopathy and burnout (nonsignificant, specific indirect effects). Another potentially surprising result is that a higher level of psychopathy among recruiters was generally associated with a slightly higher level of burnout than two other Dark Triad traits separately. In general, people who score high on psychopathy are more emotionally indifferent, and, as a consequence, feel less stress, anxiety, and exhaustion. However, it seems that working as a recruiter may prove challenging for people who score highly in psychopathy. Being in the middle of a social network of clients and managers, HR department specialists must possess the ability to adjust to social norms, rules, and scripts. If people scoring high in psychopathy cannot naturally adjust to these social contexts, they must control themselves so as not to “push the limits,” which may cause exhaustion and disengagement.

Similar to recruiters who score high in psychopathy, Machiavellians also find motivation mostly through instrumental forms of motivation, and experience more burnout symptoms than do narcissists. It is worth noting, however, that what distinguishes them from the other two Dark Triad groups is that higher instrumental motivation leads to greater levels of exhaustion. This result could be explained by the manner in which the work of the recruiter is organized: salaries are often based on bonuses for acquiring professionals; so owing to instrumental motivation (need for the bonus), Machiavellians immerse themselves in work (accept more projects and tasks), leading to greater exhaustion as they work harder than others in order to obtain rewards. Nevertheless, this process does not make them more disengaged from work. Recruiters high in Machiavellianism also share a trait with those who score highly in narcissism and psychopathy: they motivate themselves by seeking fun at work. One possible explanation is that the work of a recruiter gives them the opportunity to offer something that others desire. Being in a position of giving something to others may, in turn, afford these recruiters a certain amount of power, which might prove appealing for people who want to be flattered and use skills of persuasion and manipulation.

A more general conclusion is that when measuring the Dark Triad, it is advisable to investigate different forms of motivation and burnout in different work environments. Although our respondents who scored highly in Dark Triad traits tended to motivate themselves through external rewards, as additionally demonstrated in previous studies, the specific environment (HR recruitment) caused different forms of motivation to emerge, especially concerning people who score highly in narcissism.

We would like to emphasize the fact that the Dark Triad traits investigated in this study were of a subclinical form. Essentially, this means that each of us can exhibit some symptoms of the Dark Triad, but this does not mean that we have personality disorders. In fact, and perhaps unfortunately, given our conclusions, some Dark Triad traits (for example, high motivation related to narcissism) could even prove beneficial in the workplace.

## Limitations

One limitation of our study is that although our sample contained a sufficient number of participants, we would have preferred more. The cross-sectional results also do not allow us to draw any conclusions regarding the dynamics between the examined constructs such as the relationship between the Dark Triad traits, motivations, and burnout shaped by the time factor. We are additionally unable to formulate causality-based conclusions.

Furthermore, our study was conducted in a so-called “WEIRD” country, an acronym referring to Western, educated, industrial, rich, and democratic, which serves as a descriptor for the participants’ setting (Haidt, 2012). To the extent that most studies are conducted in such settings, one cannot deny that pan-cultural aspects are not taken into consideration here, even though they are most certainly worthy of investigation in the future. This could be of crucial importance because narcissism (for example) takes on slightly different forms in collectivistic cultures compared to in individualistic societies, with the former placing “my group” at the center of attention rather than having “me” as the key concept.

The study presented here provides us with findings to help understand the dynamics of burnout, the Dark Triad traits and motivational sources. However, it does not paint a complete picture, and thus should be treated as a prologue or first step toward developing a fuller understanding of the mechanisms underlying the relationship between the Dark Triad personality features and occupational health.

## Ethics Statement

The study did not require the approval of the ethics committee. However, we followed ethical rules in the form recommended by universities in Poland. Our subjects were informed about the nature and aim of the study. All subjects had to agree to participate in the study. We did not collect any data allowing to identify our subjects. The participants in the study were informed about possible feedback.

## Author Contributions

MP helped in substantial contribution including main body text (Introduction, Method, Results, Discussion) and data analysis and presentation. MS helped in substantial contribution including main body text (Introduction and Discussion).

### Conflict of Interest Statement

The authors declare that the research was conducted in the absence of any commercial or financial relationships that could be construed as a potential conflict of interest.

## References

[ref1] Amernic J. H. Craig R. J. (2010). Accounting as a facilitator of extreme narcissism. J. Bus. Ethics 96, 79–93. 10.1007/s10551-010-0450-0

[ref2] Ames D. (2009). Pushing up to a point: assertiveness and effectiveness in leadership and interpersonal dynamics. Res. Organ. Behav. 29, 111–133. 10.1016/j.riob.2009.06.010

[ref3] Baka Ł. (2011). Konflikt między pracą i rodziną a wypalenie zawodowe. Pośrednicząca rola zasobów osobowych. Psychologia Społeczna 6, 367–374.

[ref4] Baka Ł. Basińska B. A. (2016). Psychometryczne właściwości polskiej wersji oldenburskiego kwestionariusza wypalenia zawodowego (OLBI). Med. Pr. 67, 29–41. 10.13075/mp.5893.00353, PMID: 27044717

[ref5] Baka Ł. Cieślak R. (2010). Zależności miedzy stresorami w pracy a wypaleniem zawodowym i zaangażowaniem w prace w grupie nauczycieli: pośrednicząca rola przekonań o własnej skuteczności i wsparcia społecznego. Studia Psychologiczne (Psychol. Stud.) 48, 5–19.

[ref7] Barbuto J. E. Jr. (2001). An alternative scoring method for the motivation sources inventory: a case for ratio analysis. Psychol. Rep. 88, 385–386. 10.2466/pr0.2001.88.2.385 11351876

[ref8] Barbuto J. E. Jr. Scholl R. W. (1998). Motivation sources inventory: development and validation of new scales to measure an integrative taxonomy of motivation. Psychol. Rep. 82, 1011–1022.

[ref9] Barnett M. D. Flores J. (2016). Narcissus, exhausted: self-compassion mediates the relationship between narcissism and school burnout. Personal. Individ. Differ. 97, 102–108. 10.1016/j.paid.2016.03.026

[ref10] Bartol C. R. Bergen G. T. Volckens J. S. Knoras K. M. (1992). Women in small-town policing: job performance and stress. Crim. Justice Behav. 19, 240–259.

[ref11] Becker J. A. Dan O’Hair H. (2007). Machiavellians’ motives in organizational citizenship behavior. J. Appl. Commun. Res. 35, 246–267. 10.1080/00909880701434232

[ref12] Beutler L. E. Nussbaum P. D. Meredith K. E. (1988). Changing personality patterns of police officers. Prof. Psychol. Res. Pract. 19, 503–507. 10.1037/0735-7028.19.5.503

[ref13] Birkás B. Gács B. Csathó Á. (2016). Keep calm and don’t worry: different Dark Triad traits predict distinct coping preferences. Personal. Individ. Differ. 88, 134–138. 10.1016/j.paid.2015.09.007

[ref14] Christie R. Lehman S. (1970). “The structure of Machiavellian orientations” in Studies in Machiavellianism. eds. Christie R. Geis F. (New York: Academic), 359–387.

[ref15] Cohen A. (2016). Are they among us? A conceptual framework of the relationship between the Dark Triad personality and counterproductive work behaviors (CWBs). Hum. Resour. Manag. Rev. 26, 69–85. 10.1016/j.hrmr.2015.07.003

[ref16] Cresswell S. L. Eklund R. C. (2005). Motivation and burnout among top amateur rugby players. Med. Sci. Sports Exerc. 37, 469–477. 10.1080/02640410701784525 15741847

[ref18] Demerouti E. Bakker A. B. Nachreiner F. Schaufeli W. B. (2001). The job demands-resources model of burnout. J. Appl. Psychol. 86, 499–512. 10.1037/0021-9010.86.3.499, PMID: 11419809

[ref19] Demerouti E. Bakker A. B. Vardakou I. Kantas A. (2003). The convergent validity of two burnout instruments: a multitrait-multimethod analysis. Eur. J. Psychol. Assess. 19, 12–13. 10.1027//1015-5759.19.1.12

[ref20] Fernet C. Chanal J. Guay F. (2017). What fuels the fire: job-or task-specific motivation (or both)? On the hierarchical and multidimensional nature of teacher motivation in relation to job burnout. Work Stress 31, 145–163. 10.1080/02678373.2017.1303758

[ref21] Furnham A. Trickey G. Hyde G. (2012). Bright aspects to dark side traits: dark side traits associated with work success. Personal. Individ. Differ. 52, 908–913. 10.1016/j.paid.2012.01.025

[ref22] Haidt J. (2012). The righteous mind: Why good people are divided by politics and religion. (New York: Vintage Books).

[ref23] Hare R. D. Neumann C. S. (2009). Psychopathy: assessment and forensic implications. Can. J. Psychiatry 54, 791–802. 10.1177/070674370905401202, PMID: 20047718

[ref24] Harrison A. Summers J. Mennecke B. (2016). The effects of the Dark Triad on unethical behavior. J. Bus. Ethics 153, 53–77. 10.1007/s10551-016-3368-3

[ref25] Heisler W. J. Gemmill G. R. (1977). Machiavellianism, job satisfaction, job strain, and upward mobility: some cross-organizational evidence. Psychol. Rep. 41, 592–594.

[ref26] Johnson V. A. Beehr T. A. O’Brien K. E. (2015). Determining the relationship between employee psychopathy and strain: does the type of psychopathy matter? Int. J. Stress Manag. 22, 111–136. 10.1037/a0038817

[ref27] Jonason P. K. Ferrell J. D. (2016). Looking under the hood: the psychogenic motivational foundations of the Dark Triad. Personal. Individ. Differ. 94, 324–331. 10.1016/j.paid.2016.01.039

[ref28] Jonason P. K. Slomski S. Partyka J. (2012). The Dark Triad at work: how toxic employees get their way. Personal. Individ. Differ. 52, 449–453. 10.1016/j.paid.2011.11.008

[ref29] Jonason P. K. Webster G. D. (2012). A protean approach to social influence: Dark Triad personalities and social influence tactics. Personal. Individ. Differ. 52, 521–526. 10.1016/j.paid.2011.11.023

[ref30] Jonason P. K. Wee S. Li N. P. (2015). Competition, autonomy, and prestige: mechanisms through which the Dark Triad predict job satisfaction. Personal. Individ. Differ. 72, 112–116. 10.1016/j.paid.2014.08.026

[ref31] Jonason P. K. Wee S. Li N. P. Jackson C. (2014). Occupational niches and the Dark Triad traits. Personal. Individ. Differ. 69, 119–123. 10.1016/j.paid.2014.05.024

[ref32] Jones D. N. (2014). Risk in the face of retribution: psychopathic individuals persist in financial misbehaviour among the Dark Triad. Personal. Individ. Differ. 67, 109–113. 10.1016/j.paid.2014.01.030

[ref33] Jones D. N. Paulhus D. L. (2014). Introducing the Short Dark Triad (SD3): a brief measure of dark personality traits. Assessment 21, 28–41. 10.1177/1073191113514105 24322012

[ref34] Kernberg O. F. (1989). The narcissistic personality disorder and the differential: diagnosis of antisocial behavior. Psychiatr. Clin. 12, 553–570. 2678022

[ref35] Kessler S. R. Bandelli A. C. Spector P. E. Borman W. C. Nelson C. E. Penney L. M. (2010). Re-examining Machiavelli: a three-dimensional model of Machiavellianism in the workplace. J. Appl. Soc. Psychol. 40, 1868–1896. 10.1111/j.1559-1816.2010.00643.x

[ref36] Lee R. T. Ashforth B. E. (1996). A meta-analytic examination of the correlates of the three dimensions of job burnout. J. Appl. Psychol. 81, 123–133. 10.1037/0021-9010.81.2.123, PMID: 8603909

[ref37] Leonard N. H. Beauvais L. L. Scholl R. W. (1995). “A self-concept-based model of work motivation” in Academy of management proceedings. ed. Manor B. (NY: Academy of Management), 322–326.

[ref38] Li C. Wang C. J. Kee Y. H. (2013). Burnout and its relations with basic psychological needs and motivation among athletes: a systematic review and meta-analysis. Psychol. Sport Exerc. 14, 692–700. 10.1016/j.psychsport.2013.04.009

[ref39] Lonsdale C. Hodge K. Rose E. (2009). Athlete burnout in elite sport: a self-determination perspective. J. Sports Sci. 27, 785–795. 10.1080/02640410902929366, PMID: 19437185

[ref40] Malanowska D. M. Prusik M. (2016). Dark Triad, sources of motivation, and burnout among HR recruitment workers. Unpublished database. (Warsaw, Poland: The Maria Grzegorzewska University).

[ref42] Morf C. C. Rhodewalt F. (2001). Unraveling the paradoxes of narcissism: a dynamic self-regulatory processing model. Psychol. Inq. 12, 177–196. 10.1207/S15327965PLI1204_1

[ref44] Noser A. E. Zeigler-Hill V. Besser A. (2014). Stress and affective experiences: the importance of dark personality features. J. Res. Pers. 53, 158–164. 10.1016/j.jrp.2014.10.007

[ref45] O’Boyle E. H. Jr. Forsyth D. R. Banks G. C. McDaniel M. A. (2012). A meta-analysis of the Dark Triad and work behavior: a social exchange perspective. J. Appl. Psychol. 97, 557–579. 10.1037/a0025679, PMID: 22023075

[ref46] Palmer J. C. Komarraju M. Carter M. Z. Karau S. J. (2017). Angel on one shoulder: can perceived organizational support moderate the relationship between the Dark Triad traits and counterproductive work behavior? Personal. Individ. Differ. 110, 31–37. 10.1016/j.paid.2017.01.018

[ref47] Paulhus D. L. Williams K. M. (2002). The Dark Triad of personality: narcissism, Machiavellianism, and psychopathy. J. Res. Pers. 36, 556–563. 10.1016/S0092-6566(02)00505-6

[ref48] Paunonen S. V. Lonnquvist J.-E. Verkasalo M. Leikas S. Nissinen V. (2006). Narcissism and emergent leadership in military cadets. Leadersh. Q. 17, 475–486. 10.1016/j.leaqua.2006.06.003

[ref49] Pilch I. Górnik-Durose M. E. (2016). Do we need “dark” traits to explain materialism? The incremental validity of the Dark Triad over the HEXACO domains in predicting materialistic orientation. Personal. Individ. Differ. 102, 102–106. 10.1016/j.paid.2016.06.047

[ref50] Rauthmann J. F. Kolar G. P. (2013). The perceived attractiveness and traits of the Dark Triad: narcissists are perceived as hot, Machiavellians and psychopaths not. Personal. Individ. Differ. 54, 582–586. 10.1016/j.paid.2012.11.005

[ref51] Rauthmann J. F. Will T. (2011). Proposing a multidimensional Machiavellianism conceptualization. Soc. Behav. Pers. 39, 391–403. 10.2224/sbp.2011.39.3.391

[ref52] Rawolle M. Wallis M. S. Badham R. Kehr H. M. (2016). No fit, no fun: the effect of motive incongruence on job burnout and the mediating role of intrinsic motivation. Personal. Individ. Differ. 89, 65–68. 10.1016/j.paid.2015.09.030

[ref53] Richardson E. N. Boag S. (2016). Offensive defenses: the mind beneath the mask of the Dark Triad traits. Personal. Individ. Differ. 92, 148–152. 10.1016/j.paid.2015.12.039

[ref54] Roczniewska M. Bakker A. B. (2016). Who seeks job resources, and who avoids job demands? The link between dark personality traits and job crafting. J. Psychol. 150, 1026–1045. 10.1080/00223980.2016.1235537 27719551

[ref55] Rosenthal S. A. Pittinsky T. L. (2006). Narcissistic leadership. Leadersh. Q. 17, 617–633. 10.1016/j.leaqua.2006.10.005

[ref17] Ryan R. M. Deci E. L. (2002). “Overview of self-determination theory: An organismic-dialectical perspective” in Handbook of self-determination research. eds. Deci E. L. Ryan R. M. (Rochester, NY, US: University of Rochester Press), 3–33.

[ref56] Schaufeli W. B. Bakker A. B. (2004). Job demands, job resources, and their relationship with burnout and engagement: a multi-sample study. J. Organ. Behav. 25, 293–315. 10.1002/job.248

[ref57] Schwarzkopf K. Straus D. Porschke H. Znoj H. Conrad N. Schmidt-Trucksäss A. (2016). Empirical evidence for a relationship between narcissistic personality traits and job burnout. Burn. Res. 3, 25–33. 10.1016/j.burn.2015.12.001

[ref58] Van den Berghe L. Soenens B. Aelterman N. Cardon G. Tallir I. B. Haerens L. (2014). Within-person profiles of teachers’ motivation to teach: associations with need satisfaction at work, need-supportive teaching, and burnout. Psychol. Sport Exerc. 15, 407–417. 10.1016/j.psychsport.2014.04.001

[ref59] Van den Broeck A. Vansteenkiste M. De Witte H. Lens W. (2008). Explaining the relationships between job characteristics, burnout, and engagement: the role of basic psychological need satisfaction. Work Stress 22, 277–294. 10.1080/02678370802393672

[ref60] Volmer J. Koch I. K. Göritz A. S. (2016). The bright and dark sides of leaders’ Dark Triad traits: effects on subordinates’ career success and well-being. Personal. Individ. Differ. 101, 413–418. 10.1016/j.paid.2016.06.046

