# Supplementary figures and images for: The Relationship Between the Dark Triad Personality Traits, Motivation at Work, and Burnout Among HR Recruitment Workers
Source: Front Psychol. 2019 Jun 7;10:1290. doi: 10.3389/fpsyg.2019.01290 (PMC6566539; doi:10.3389/fpsyg.2019.01290)

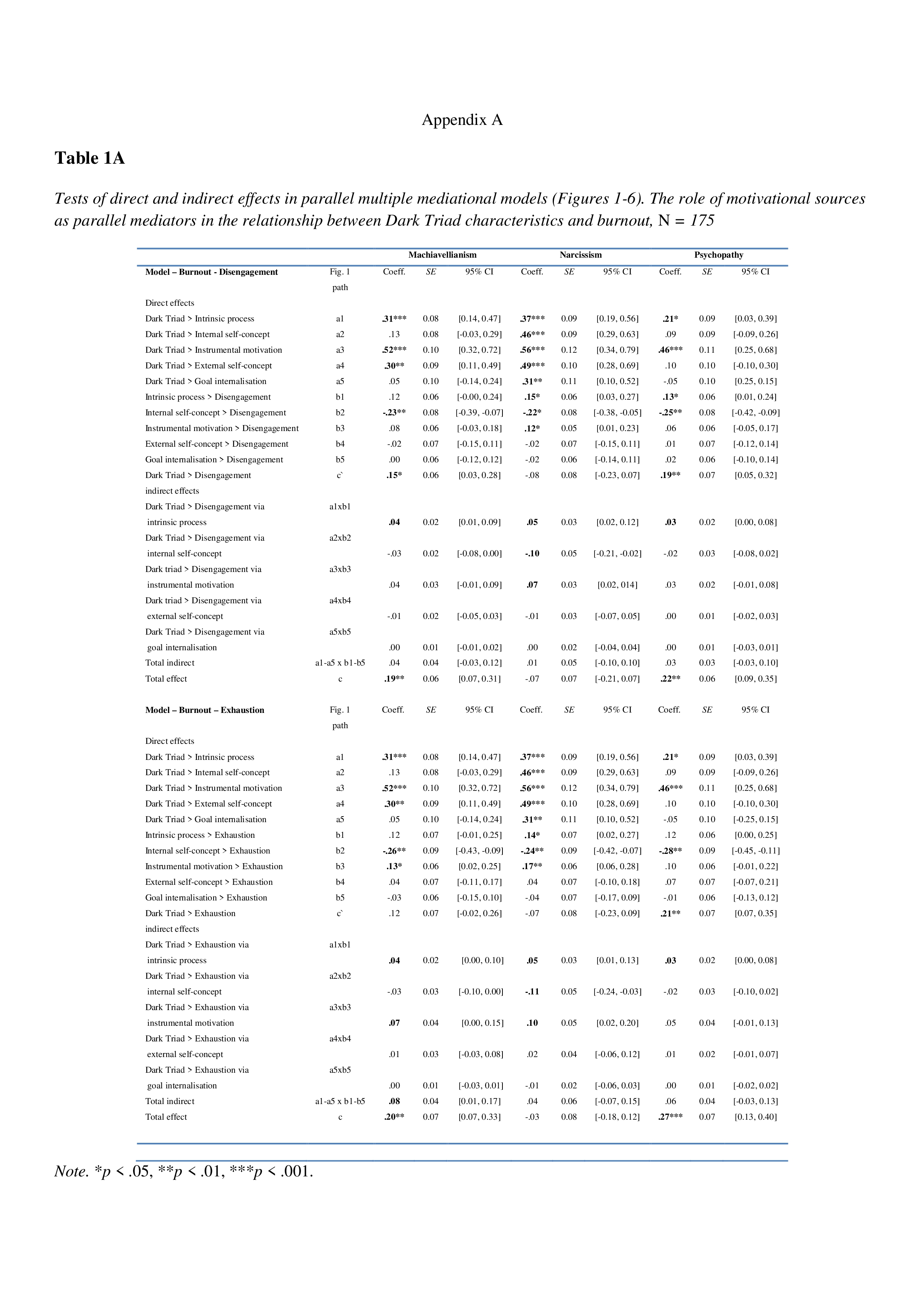

Supplement: Supplementary file 1 [file Image_1.JPEG]
